# Topical CCL3 Is Well-Tolerated and Improves Liver Function in Diabetic Mice: Evidence from a 14-Day Toxicity Study

**DOI:** 10.3390/cells15020120

**Published:** 2026-01-09

**Authors:** Deepa Dehari, Rajalekshmy Padmakumari, Getnet Tesfaw, Fernando A. Fierro, Guillermo A. Ameer, Sasha H. Shafikhani

**Affiliations:** 1 Department of Dermatology, University of California Davis School of Medicine, Sacramento, CA 95817, USA; 2 Stem Cell Program, Department of Internal Medicine, University of California Davis, Sacramento, CA 95817, USA; 3 Department of Biomedical Engineering, Northwestern University, Evanston, IL 60208, USA; 4 Department of Surgery, Feinberg School of Medicine, Northwestern University, Chicago, IL 60611, USA; 5 Querrey Simpson Institute for Regenerative Engineering at Northwestern University, Chicago, IL 60611, USA

**Keywords:** diabetic mice, wound healing, CCL3 (MIP-1α), acute toxicity, histopathology, biochemical analysis, immunomodulators, wound care

## Abstract

Diabetic wounds exhibit impaired immune function, delayed neutrophils recruitment, and heightened infection risk which compromises early infection control and delays healing. We have demonstrated that topical CCL3 treatment restores neutrophil influx, reduces bacterial infection by ~99%, and accelerates wound healing in diabetic mice. As per Food and Drug Administration (FDA) Guidelines for Investigational New Drug (IND), we conducted a 14-day acute toxicity study in diabetic mice following a single topical administration of CCL3 at effective low dose (1 µg) and high dose (10 µg) per wound. Mice were monitored for clinical signs, body weight, and food intake throughout the study period. On day 14, serum biochemistry (ALT, AST, BUN, creatinine, metabolic markers) and histopathology of major organs (liver, kidney, heart, lungs, spleen) were assessed. CCL3-treated diabetic mice exhibited no adverse clinical effects. Hematological and biochemical parameters remained within normal limits, and histopathological analyses revealed no additional organ injury in CCL3-treated groups compared to diabetic control mice. Intriguingly, CCL3-treated mice showed improved ALT levels and reduced hepatic pathology, suggesting hepatoprotective effects and reduced serum IgG, indicating reduced systemic inflammation. Overall, our study demonstrates that diabetic mice tolerate topical CCL3 at doses up to 10 times the effective therapeutic concentration without evidence of systemic organ toxicity. These findings provide strong preclinical support for the translational development of CCL3 as a novel therapy for diabetic wound care.

## 1. Introduction

Diabetic foot ulcers (DFUs) are one of the most prevalent and debilitating consequences of diabetes, which account for 60–75.5% of all lower extremity amputations (LEA) and cost ~$9–12 billion in the annual care in the United States alone [1]. The staggering number of ulcers that progress to LEA underscores the inadequacy of current treatments and the urgent need for innovative approaches. Infection is a major co-morbidity and a driver of chronic state and LEA in DFUs [1]. Unlike chronic ulcers that are locked in a state of persistent and non-resolving inflammation [2,3], inflammatory responses are significantly diminished during the acute phase of healing early after injury, making diabetic wounds susceptible to infection with pathogenic bacteria such as *Pseudomonas aeruginosa* which further exacerbate tissue damage and impair tissue repair through the production of their virulence factors that can further dampen inflammatory responses and adversely affect a multitude of cellular processes underlying tissue repair [4,5,6,7,8,9,10,11].

Neutrophils are the first line of defense against invading pathogens, immediately migrating to the site of injury to facilitate bacterial clearance by phagocytosis, the generation of reactive oxygen and nitrogen species, antimicrobial peptides, and the development of neutrophil extracellular traps [12,13]. However, antimicrobial and bactericidal functions are impaired in neutrophils under hyperglycemia and diabetic conditions, rendering them ineffective to control infection [14]. In addition, neutrophils isolated from the blood of diabetic patients exhibit impaired chemotactic responses, leading to reduced neutrophil trafficking into the wound and toward infection during the early stages of diabetic wound healing. This neutrophil dysfunction is attributed to impaired signaling through the formyl peptide receptor (FPR), which normally mediates the initial wave of neutrophil trafficking to injury and infection [4,15]. Importantly, some auxiliary chemokine receptors, such as CCR1, remain functional under diabetic conditions; however, they are not activated in diabetic wounds in the early stages due to low expression of their endogenous ligands [4,6].

There are currently no approved biologic therapies that target infection in diabetic wounds. The only approved biologic therapy for DFUs is Becaplermin gel (0.01% rhPDGF-BB). However, Becaplermin has limited efficacy in stimulating healing and is not effective against infection [16,17]. Antibiotics are routinely included in the management of diabetic ulcers [18,19], but they are linked to emergence of antibiotic resistance, toxicity, and interference with tissue repair [20,21,22]. These reports underscore the urgent need for novel therapeutics (preferably antibiotic-free) to address infection in diabetic wounds. We have previously demonstrated that topical treatment with CCL3/MIP-1α immunomodulator reduced infection by >99% and improved healing in diabetic mice by bypassing impaired FPR signaling and restoring timely neutrophil trafficking, highlighting its potential in diabetic wound care [4]. However, regulatory approval of novel biologics requires comprehensive evaluation of their efficacy and toxicity in relevant animal models [23,24].

The present study investigated the 14-day toxicity profile of topical CCL3 wound therapy in diabetic mice, including physiological monitoring, biochemical and hematological analyses, and histopathological assessment of major organs. The findings offer fundamental insights into immunoloffgical safety and systemic acceptability of CCL3 treatment in diabetic wound models, paving the way to clinical applications.

## 2. Material and Methods

### 2.1. Chemicals

Phosphate buffer saline (PBS) pH 7.4 (Gibco™, Life Technologies Corporation, Carlsbad, CA, USA), recombinant mouse CCL3/mip-1 alpha protein (Biotechne/R&D systems, Minneapolis, MN, USA), ketamine hydrochloride (Dechra, Overland Park, KS, USA), xylazine (Rampun^®^, Dechra, Overland Park, KS, USA), buprenorphine (NewYork, NY, USA). All the anesthetic agent and analgesic agents procured through UC Davis MWI veterinary supply. Sterile alcohol prep pads (Fisherbrand^TM^, Pittsburgh, PA, USA), 5 mm biopsy punch (Acuderm, inc, Fort Lauderdale, FL, USA), 10% neutral buffered formalin (Epredia™, Kalamazoo, MI, USA), xylene (MerckMillipore, St. Louis, MO, USA), hematoxylin (Epredia™, Kalamazoo, MI, USA), eosin (Epredia™, Kalamazoo, MI, USA), Invitrogen mouse CRP ELISA kit (Life Technologies Corporation, Carlsbad, CA, USA), Invitrogen mouse IgG1 uncoated ELISA kit (Life Technologies Corporation, Carlsbad, CA, USA), Invitrogen mouse IgE ELISA kit (Life Technologies Corporation, Carlsbad, CA, USA).

### 2.2. Animals and Grouping

We used 8-week-old B6.BKS(D)-Lepa*^rd^*/J (db/db) obese diabetic mice (strain #00697; The Jackson Laboratory), including both sexes (3 males and 3 females per group), as the animal model for this study [25]. Age-matched heterozygous (db/+) mice of both sexes served as normoglycemic controls. All animals were obtained from The Jackson Laboratory and housed in the University of California Davis Health Animal Vivarium. Mice were maintained under controlled environmental conditions (temperature: 22 ± 2 °C; relative humidity: 50 ± 10%) with a 12 h light/dark cycle at ad libitum access to standard chow and water. All experimental procedures were conducted in accordance with the guidelines of the Institutional Animal Care and Use Committee (IACUC, Protocol #24063).

**Figure 1:**
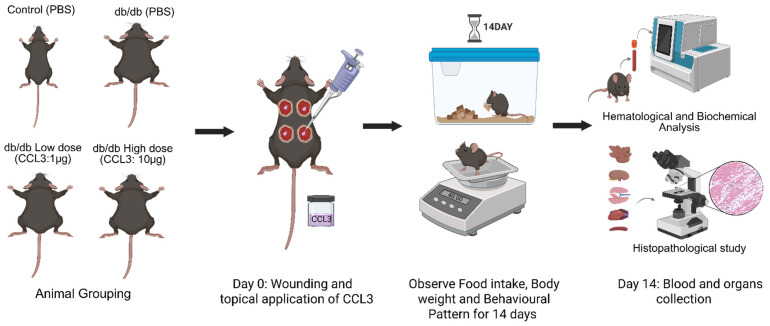
Experimental design of the toxicity study. Animals were assigned to control and db/db groups (male and female) receiving PBS or topical CCL3 at low (1 µg) or high (10 µg) doses. On day 0, full-thickness wounds were created and CCL3 or PBS was applied topically. Animals were monitored daily for food intake, body weight, and behavioral changes for 14 days. At the end of the study period, blood and major organs were collected for hematological, biochemical, and histopathological analyses. (Created in BioRender. DEHARI, D. (2026) https://BioRender.com/urf38ln).

Mice were surgically wounded by 5 mm sterile biopsy punches as described [5,26]. They were then randomly assigned to four experimental groups (6 mice/group): (1) control (db/+ receiving PBS); (2) diabetic (db/db receiving PBS); (3) diabetic treated with CCL3 at low dose (1 µg per wound); And (4) diabetic treated with CCL3 at high dose (10 µg per wound). The schematic diagram of the research strategy is depicted in Figure 1. All wounds in the same mouse received the same treatment to prevent immune confusion that has been reported [27].

### 2.3. Wounding and Treatment

One day prior to the wounding (day-1), the dorsal hair was removed with an electric trimmer to expose the skin surface. On day 0, mice were anesthetized with an intraperitoneal injection of ketamine (80 mg/kg) and xylazine (10 mg/kg), followed by analgesia with buprenorphine (0.01 mg/kg) for pain management. The dorsal skin was sanitized by wiping with a sterile alcohol pre pad, and full-thickness excisional wounds (5 mm diameter) were made using a sterile biopsy punch. Following wounding, PBS or CCL3 (1 µg or 10 µg per wound) was applied topically based on group allocation. Mice were divided into control and treatment groups, wounded, and then treated with PBS or CCL3 topically (Figure 1). They were also observed every day for changes in behavior, body weight, and food consumption. To ascertain the systemic safety profile of CCL3 treatment, main organs were histologically evaluated, and blood was studied for hematological and biochemical markers at the end of the trial.

### 2.4. Monitoring and Clinical Observations

Following treatment, mice were observed for survival, movement, posture, and any signs of discomfort. Body weight and food intake were measured at the start of the trial and at regular intervals during the 14-day period. Daily observations were also made to note any behavioral changes as described [28] that could suggest systemic toxicity associated with CCL3 treatment.

### 2.5. Immunological, Hematological, and Biochemical Analysis

To assess the systemic safety profile of CCL3 treatment in diabetic mice, immunological, hematological, and biochemical parameters were evaluated using established procedures [29]. On the 14-day post-injury and treatment, the mice were anesthetized, and blood was collected via cardiac puncture. For plasma collection, blood was drawn into BD Vacutainer™ (Becton, Dickinson and Company, Franklin Lakes, NJ, USA) collection tubes containing EDTA as an anticoagulant and centrifuged at 1000× *g* for 10 min at 8 °C, after which the supernatant plasma was collected. For serum separation, blood was collected in BD Microtainer™ serum separator tubes with gel, allowed to clot at room temperature for 30 min, and centrifuged at 4000× *g* for 10 min at room temperature to obtain serum [29]. Plasma and serum samples were prepared and sent to the Comparative Pathology Laboratory at the University of California Davis, School of Veterinary Medicine for extensive hematological and biochemical testing. Red and white blood cell counts (RBC and WBC), hemoglobin (Hb), platelet counts, and differential leukocyte distribution (monocyte, eosinophil, and basophil) were all analyzed using an automated hematology analyzer (Hemavet 950FS, Boule Diagnostics AB, Spånga, Sweden). The serum biochemistry was analyzed by Roche Cobas Integra 400 Plus (Roche Diagnostics, Basel, Switzerland), for indicators of liver and kidney functions including, Alanine Transaminase (ALT), Aspartate Aminotransferase (AST), Alkaline Phosphatase (ALP), total bilirubin, Blood Urea Nitrogen (BUN), creatinine, protein levels (total protein and albumin), electrolytes, and glucose as described [30].

Furthermore, serum samples were analyzed for immunological parameters, including inflammatory and allergy markers such as, C-reactive protein (CRP), Immunoglobulin G1 (IgG1), and Immunoglobulin E (IgE), using enzyme-linked immunosorbent assay (ELISA) methodology.

### 2.6. Histopathological Analysis

Histopathological examination was used as a gold-standard method to visualize and confirm whether CCL3 treatment altered tissue architecture or caused signs of organ toxicity, providing direct insight beyond hematological and biochemical measurements. Following blood collection, the mice were euthanized, and key organs such as the liver, kidney, spleen, heart, and pancreas were taken and fixed in 10% neutral buffered formalin. The organs were processed, paraffin-embedded, sectioned at 4–5 µm, and stained with Hematoxylin & Eosin (H&E). All histological slides were coded, and scoring was performed blinded to treatment group allocation. Histological evaluation was performed under light microscopy, and semi-quantitative scoring was utilized to quantify necrosis, inflammation, and tissue structure [31]. Semi-quantitative scoring for necrosis and inflammation was performed according to previously published criteria [32]. Necrosis was graded on a scale of 0–5: 0 = no necrosis, 1 = minimal (<5% tissue), 2 = mild (5–20%), 3 = moderate (21–40%), 4 = severe (41–60%), and 5 = very severe (>60%). Inflammation was similarly graded on a scale of 0–5: 0 = no infiltrates, 1 = minimal infiltration (few scattered cells), 2 = mild (<10% tissue), 3 = moderate (10–30%), 4 = severe (31–50%), and 5 = very severe (>50%).

### 2.7. Statistical Analysis

Statistical analyses between groups were performed using One-way ANOVA with appropriate post hoc testing. Data are presented as mean ± SEM, and *p*-values ≤ 0.05 were considered statistically significant. Prior to parametric analysis, assumptions of normality and homogeneity of variance were evaluated for all datasets using GraphPad Prism (version 10.4.2). Normality was assessed with the Shapiro–Wilk test (*n* < 30), and homogeneity of variance was evaluated using the F-test for two-group comparisons and the Brown–Forsythe test for comparisons involving more than two groups. No violations of these assumptions were detected.

## 3. Results

The experimental design is illustrated in Figure 1. Briefly, mice were surgically wounded using 5 mm sterile biopsy punches as previously described [5,26,33]. They were then randomly assigned to four experimental groups (6 mice per group): (1) control (db/+ receiving PBS); (2) diabetic (db/db receiving PBS); (3) diabetic treated with low-dose CCL3 (1 µg per wound); and (4) diabetic treated with high-dose CCL3 (10 µg per wound). Food intake, body weight, and any signs of mortality or behavioral changes were monitored over a 14-day period. On day 14, mice were euthanized, and their blood and major organs were collected for biochemical, immunological, and histological analyses to evaluate the systemic effects of CCL3 treatment on overall health.

### 3.1. Physiological and Behavioral Monitoring Following CCL3 Treatment

**Figure 2:**
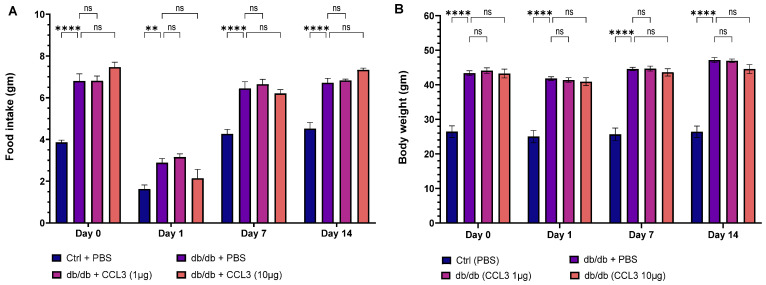
Effects of CCL3 treatment on food intake and body weight in diabetic mice. (**A**) Food intake was measured on Days 0, 1, 7, and 14 in nondiabetic (db/+) mice treated with PBS and diabetic (db/db) mice treated with PBS, CCL3 (1 µg), or CCL3 (10 µg). db/db mice exhibited significantly increased food intake at Days 7 and 14 compared with db/+ controls, with no significant differences observed among db/db treatment groups. (**B**) Body weight was monitored following wound surgery and treatment at the indicated time points. db/db mice remained significantly heavier than db/+ controls throughout the study, and CCL3 treatment had no effect on body weight. Data are presented as mean ± SEM. Statistical analysis was performed using one-way ANOVA with multiple-comparison post hoc testing (*n* = 6 mice per group; not significant differences ns; Significance differences represent as ** *p* < 0.01 **** *p* < 0.0001).

Following wound surgery on day 0, mice in all groups showed reduced mobility and decreased grooming behavior, which is consistent with acute post-surgical discomfort [34,35]. However, by day 2, behavioral patterns had recovered, and the animals resumed normal movement and feeding, with no further abnormalities noted. Nondiabetic control mice (db/+) consumed about 3–4 g of food per day, whereas diabetic mice (db/db) consumed significantly higher amounts (6.5–8 g per day), which is consistent with increased appetite in db/db obese diabetic mice [36]. Of note, food intake was temporarily reduced in all groups following surgery on days 0 and 1, but this reduced appetite for food was temporary, and by day 2 the animals had resumed their normal eating behavior (Figure 2A). Compared to db/+ nondiabetic control mice, db/db diabetic mice continued to consume more food throughout the duration of this study regardless of their treatments. PBS-treated and CCL3-treated db/db mice did not differ significantly at either dose, indicating that topical CCL3 at 1 µg and 10 µg had no effect on feeding behavior. Throughout the study, db/db mice in all groups weighed significantly more (40–50 g) than nondiabetic db/+ mice (20–30 g) controls at baseline (day 0) (*p* < 0.0001). Despite loss of appetite on day 1, mice did not experience any significant weight loss following wounding procedure and their weights remained constant throughout this 14-day period (Figure 2B). Notably, the db/db groups treated with PBS and CCL3 did not differ significantly in their eating behavior or weight, indicating that CCL3 treatments were well tolerated and did not change metabolic status in mice (Figure 2B).

### 3.2. CCL3 Therapy Immunological Assessment in Diabetic Mice

We assessed serum immunological parameters, and circulating biomarkers in day 14 blood to determine whether CCL3 treatment adversely altered immune responses or caused systemic toxicity. In terms of immunological parameters associated with systemic inflammation, compared to nondiabetic control group, diabetic mice treated with PBS (mock) exhibited approximately 48.5% higher serum IgG1 levels (285.75 ± 86.27 µg/mL vs. 192.45 ± 13.51 µg/mL, *p* < 0.01); 125% higher IgE (207.35 ± 57.64 vs. 92.29 ± 22.03, *p* < 0.01); and 335% higher C-reactive protein (CRP) levels (8.14 ± 1.86 vs. 1.83 ± 0.33, *p* < 0.001) (Table 1). These findings are consistent with clinical reports showing that diabetic patients exhibit elevated serum levels of IgG1, IgE, and CRP, associated with increased risks of kidney nephropathy, allergic reactions, systemic inflammation, and diabetic retinopathy [37,38,39,40,41,42,43].

**Figure 3:**
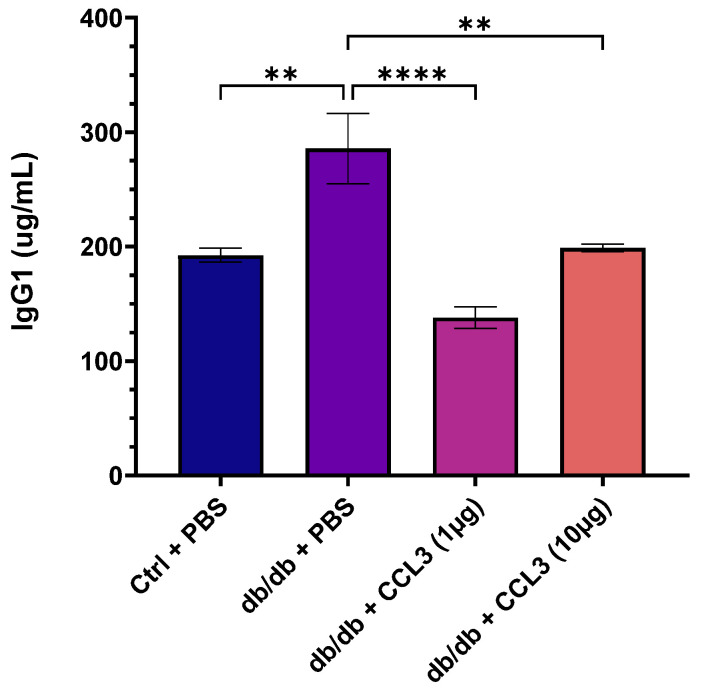
Evaluation of serum IgG1 levels as an immunological safety marker following CCL3 treatment. Serum IgG1 levels were quantified on day 14 in nondiabetic (db/+) mice treated with PBS and diabetic (db/db) mice treated with PBS, CCL3 (1 µg), or CCL3 (10 µg). Diabetic PBS-treated mice exhibited elevated IgG1 levels, indicative of systemic inflammation, whereas CCL3-treated groups showed a marked reduction, demonstrating that topical CCL3 does not elicit immunotoxic responses. Data are represented as mean ± SEM (*n* = 6 mice per group). Statistical analysis was performed using one-way ANOVA; Significance level represent as ** *p* < 0.01, **** *p* < 0.0001.

Interestingly, CCL3 treatment lowered serum IgG1 in diabetic mice to levels similar to those of nondiabetic controls (Figure 3), suggesting a beneficial CCL3-induced modulation of dysregulated adaptive immune responses that are commonly associated with diabetes [38,39,44]. IgE and CRP levels in the diabetic groups treated with CCL3 were not significantly different compared to PBS-treated diabetic group (Table 1). Collectively, these results indicate that CCL3 therapies do not adversely affect these immune biomarkers. Rather, they may potentially have a beneficial impact on serum IgG1.

### 3.3. CCL3 Therapy Biochemical Safety Assessment in Diabetic Mice

To further evaluate the systemic safety of CCL3 therapy, serum biochemical parameters were assessed on day 14. As anticipated, glucose levels were significantly higher in db/db mice (402.48 ± 34.02 mg/dL) compared with db/+ controls (231.68 ± 24.41 mg/dL); however, CCL3 treatment did not further affect serum glucose levels (Table 2). Serum electrolytes (sodium, potassium, chloride, calcium, and phosphorus), as well as total protein and bilirubin concentrations, were similar between PBS- and CCL3-treated diabetic mice, indicating that CCL3 administration did not alter these parameters (Table 2).

**Figure 4:**
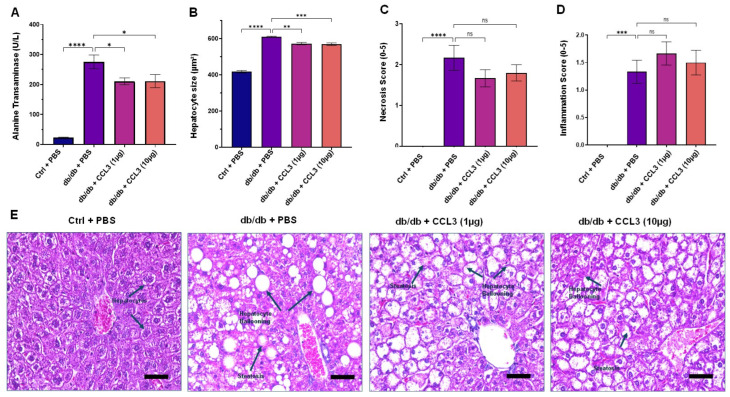
CCL3 treatment confers modest but significant hepatoprotective benefits in diabetic mice. (**A**) Serum alanine transaminase (ALT) levels, (**B**) hepatocyte size, (**C**,**D**) necrosis and inflammation scores were assessed on day 14 in control (db/+) mice treated with PBS and diabetic (db/db) mice treated with PBS, CCL3 (1 µg), or CCL3 (10 µg). (**E**) Representative H&E-stained liver sections showing normal hepatic architecture in control mice and pronounced steatosis, hepatocyte ballooning in PBS- and CCL3-treated diabetic mice. Data are presented as mean ± SEM (*n* = 6 per group). Statistical analysis was performed using one-way ANOVA followed by multiple comparisons; Significance level represent as * *p* < 0.05, ** *p* < 0.01, *** *p* < 0.001, **** *p* < 0.0001, non-significance represents as ns. Scale bars = 50 µm.

Liver enzymes—including alanine aminotransferase (ALT), aspartate aminotransferase (AST), and alkaline phosphatase (ALP)—are frequently elevated in pre-diabetic and diabetic individuals and have been associated with obesity, visceral adiposity, dyslipidemia, increased diabetes risk, and metabolic dysfunction–associated steatosis liver disease (MASLD) [45,46,47,48,49,50]. In line with these observations, by day 14 post-treatment the PBS-treated diabetic (db/db) control mice exhibited markedly elevated serum ALT (275.65 ± 22.94 U/L), AST (247.58 ± 11.97 U/L), and ALP (217.00 ± 6.32 U/L) levels compared with the nondiabetic control group (ALT: 23.27 ± 1.33 U/L; AST: 83.39 ± 6.18 U/L; ALP: 97.93 ± 16.37 U/L) (Table 2 and Figure 4A). Interestingly, CCL3- treated diabetic mice exhibited modest but significant reductions in ALT levels at both 1 µg (210.35 ± 11.43 U/L) and 10 µg (211.18 ± 22.02 U/L) compared with PBS-treated diabetic controls (*p* < 0.05; Figure 4A, and Table 2). Of note, serum AST and ALP levels were not affected by CCL3 topical treatments and remained significantly elevated in diabetic mice compared to nondiabetic mice.

To further assess the impact of CCL3 on liver injury, we performed histological analysis of hepatic tissue (Materials and Methods). Compared with nondiabetic mice, diabetic control mice (db/db + PBS) exhibited marked steatosis, hepatocyte ballooning, disruption of normal lobular architecture, and elevated necrosis and inflammation scores (Figure 4A–D). These histopathological features are consistent with MASLD and liver injury reported in patients with type 2 diabetes [51,52,53]. In contrast, mice treated with CCL3 at either dose showed reduced hepatocyte size, and trended toward reduced necrosis scores, albeit they did not reach statistical significance (Figure 4B–E). Collectively, these findings indicate that CCL3 treatment does not exacerbate liver injury and suggest a modest hepatoprotective effect in diabetic mice.

### 3.4. CCL3 Does Not Cause Organ Toxicity: Histopathological Evidence

**Figure 5:**
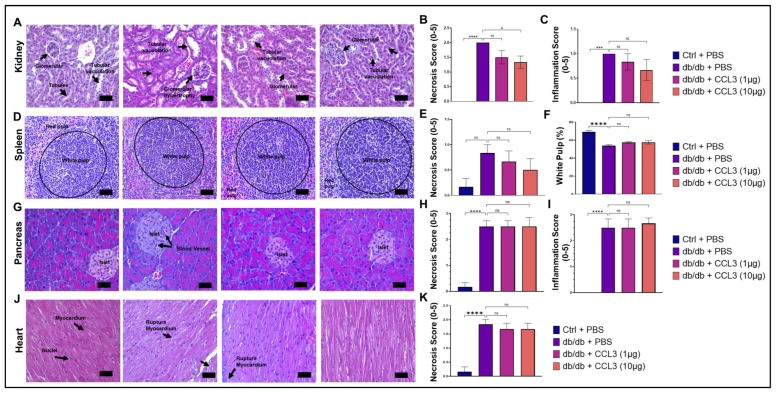
Histopathological assessment of major organs following topical CCL3 treatment. Representative H&E images of kidney (**A**–**C**), spleen (**D**–**F**), pancreas (**G**–**I**), and heart (**J**,**K**) collected on day 14 from control (db/+) mice, diabetic (db/db) mice treated with PBS, and diabetic mice treated with CCL3 (1 µg or 10 µg). Diabetic PBS mice displayed typical diabetic alterations, while CCL3-treated groups showed no additional pathology. Semiquantitative necrosis and inflammation scores (right panels) revealed no significant differences between CCL3-treated and diabetic PBS groups. Data represents as mean ± SEM (*n* = 6). One-way ANOVA followed by post hoc multiple comparison test; Significance level represent as * *p* < 0.05, *** *p* < 0.001, **** *p* < 0.0001 and, non-significance represents as ns. Scale bars = 50 µm.

Kidney, spleen, pancreas, and heart are among other important organs that are adversely affected by diabetic condition [54,55,56,57,58,59]. We performed histopathological evaluation of these organs to assess potential systemic toxicity following topical CCL3 treatment in diabetic db/db mice. Representative hematoxylin and eosin-stained sections from liver, kidney, spleen, pancreas, and heart are shown, together with semi-quantitative scoring of necrosis and inflammation (Figure 5).

Renal histology in db/db + PBS mice revealed characteristic diabetic nephropathy–like changes [54], including tubular vacuolation and glomerular hypertrophy (Figure 5A). In contrast, kidneys from CCL3-treated mice displayed comparable histological features without additional structural damage. Quantitative scoring confirmed that necrosis and inflammation levels remained similar across all treatment groups, with no evidence of worsening renal pathology following topical CCL3 application (Figure 5B,C).

Diabetes causes significant histological changes in the spleen, leading to structural alterations like white pulp atrophy, reduced lymphoid nodules/germinal centers, decreased lymphocyte density, and altered immune cell distribution, often linked to increased oxidative stress and inflammation, resulting in impaired immune function [55]. The spleen of db/+ control mice showed normal histology, with clearly demarcated white pulp (lymphocyte-rich areas) and red pulp (erythrocyte-rich areas). In contrast, diabetic PBS-treated control mice showed disrupted architecture with a lower white pulp percentage compared to non-diabetic control group (53.82 ± 2.94% vs. 69.11 ± 4.13%, *p* < 0.0001), indicating impaired immune organization (Figure 5D). CCL3-treated diabetic mice regardless of dose, showed a similar reduction in white pulp (57.33 ± 2.75% and 57.64 ± 4.9%, respectively), with no statistical differences from db/db PBS control group, indicating that treatment did not impact splenic changes (Figure 5D–G).

Diabetic patients display significant histological changes in the pancreas, especially in the islets of Langerhans, including beta-cell loss, insulitis (inflammation in Type 1), and islet amyloidosis (protein deposits in Type 2), alongside potential exocrine tissue damage like fat accumulation and fibrosis, affecting both insulin production and overall pancreatic structure and function [56,57]. As expected, nondiabetic db/+ control mice had normal islet and acinar tissue, whereas db/db control mice showed islet distortion and vascular congestion (Figure 5G). Consistent with this data, diabetic mice showed significantly higher necrosis and inflammation scores than controls (Figure 5H, I, *p* < 0.0001). Semi-quantitative scoring showed no evidence of additional CCL3-associated pancreatic toxicity (Figure 5H,I).

Cardiomyopathy, and cardiovascular problems, including focal myofiber alterations have been reported in diabetic patients [58,59]. Consistent with these reports, cardiac sections from db/db + PBS mice exhibited myocardial disruption consistent with diabetic cardiomyopathy, including focal myofiber alterations and increased necrosis scores (Figure 5J,K). Importantly, topical CCL3 treatment did not increase myocardial rupture, necrosis, or inflammatory changes at the examined doses.

Collectively, histopathological analysis across multiple organs demonstrates that topical CCL3 treatment, even at 10-fold higher dose, does not induce systemic organ toxicity or exacerbate diabetes-associated tissue pathology, supporting its favorable safety profile in this model.

### 3.5. CCL3 Therapy Hematological Assessment

To further evaluate the systemic safety of CCL3 therapy hematological parameters were assessed on day 14. White blood cell (WBC) counts were significantly elevated in db/db mice compared with their db/+ control counterparts (Table 3), a finding that is consistent with previously reported hematological alterations [60]. Importantly, administration of CCL3 at either the 1 µg or 10 µg dose did not further alter circulating WBC levels, indicating that CCL3 treatment does not exacerbate diabetes-associated leukocytosis. In addition, no significant differences were detected in hemoglobin concentrations or in the relative proportions of major leukocyte subsets, including neutrophils, lymphocytes, monocytes, eosinophils, and basophils, when comparing diabetic and nondiabetic mice or PBS-treated and CCL3-treated diabetic groups (Table 3). Consistent with these findings, platelet counts and mean corpuscular volume (MCV) remained comparable across all experimental conditions, and mean platelet volume (MPV) showed no detectable variation among groups (Table 3). Collectively, these data demonstrate that CCL3 administration at the doses tested does not induce measurable hematological abnormalities, supporting the conclusion that CCL3 therapy is not associated with hematological toxicity under these experimental conditions.

## 4. Discussion

Previously, we demonstrated that the proinflammatory cytokine CCL3 has therapeutic potential in restoring neutrophil function, improving bacterial clearance, and stimulating healing in diabetic wounds [4]. However, the lack of comprehensive safety assessment has limited its translational advancement as an investigative novel biologic for diabetic wound care. The present study is the first to evaluate the acute systemic safety profile of topical CCL3 (MIP-1α) in a diabetic wound model. Our findings show that in db/db mice, single-dose topical administration of CCL3 at the experimentally effective dose and at a 10-fold higher dose was well tolerated, with no evidence of hematological, biochemical, or histopathological toxicity in major organs. Notably, CCL3-treated mice exhibited partial normalization of systemic inflammatory markers and reduced hepatocellular injury, highlighting CCL3’s therapeutic potential and safety as a novel biologic for diabetic wound management.

Interestingly, we found that CCL3 treatment reduced serum ALT levels while partially preserving hepatic architecture, suggesting a modest attenuation of liver injury markers rather than definitive hepatoprotection. Elevated liver enzymes and fatty liver changes have been well documented in diabetic mice and patients with type 2 diabetes [61,62]. In our study, CCL3-treated mice showed reduced hepatocyte size, supporting the idea that CCL3-induced neutrophil recruitment may aid in the clearance of necrotic cells and restoration of tissue homeostasis. Similar findings have been reported for CXCL1 and other neutrophil chemokines, where increased early neutrophil infiltration accelerated bacterial clearance while reducing secondary tissue damage [63,64]. However, the observed reduction in serum ALT levels and partial improvement in liver histopathology in CCL3-treated diabetic mice should be interpreted with caution. These effects were modest, did not normalize ALT values to non-diabetic levels, and were not accompanied by changes in AST or ALP, indicating that any impact on liver injury markers is partial rather than comprehensive. In the absence of mechanistic studies demonstrating a direct hepatic action of CCL3, it is plausible that these changes reflect indirect effects, such as reduced systemic inflammation or altered immune signaling secondary to improved wound healing and immune homeostasis. As such, these findings are best viewed as preliminary and hypothesis-generating, highlighting the need for future studies to delineate the mechanisms underlying these observations and to determine their relevance to liver pathology in diabetic disease.

A major concern with any cytokine or chemokine-based therapy is the risk of systemic immune dysregulation or hypersensitivity. Overall, systemic immunological profiling supports the safety of topical CCL3 therapy in diabetic mice, as treatment did not exacerbate circulating markers, including IgE and CRP which are commonly elevated in diabetes and linked to disease complications [42,65]. Importantly, CCL3 selectively reduced serum IgG1 levels in diabetic mice to values comparable to nondiabetic controls, suggesting a normalization of dysregulated adaptive immune responses with respect to IgG1. Given that elevated IgG1 has been associated with chronic low-grade systemic inflammation and a higher risk of developing macrovascular and microvascular complications, particularly diabetic nephropathy and cardiovascular disease (CVD) [39,44,66], this selective modulation may represent a beneficial immunological effect of CCL3 treatment. Together, these findings indicate that topical CCL3 is well tolerated at the doses tested and may confer additional systemic benefit through attenuation of aberrant IgG1 elevation, while avoiding broad suppression or activation of inflammatory pathways.

Hematological and biochemical analyses have been accepted as gold-standard parameters in preclinical toxicity testing (OECD Test Guidelines; FDA Preclinical Research guidelines). In our investigation, total blood parameters and biochemistry markers remained within physiological ranges in all groups, indicating that systemic homeostasis was maintained. Notably, diabetic PBS mice demonstrated higher liver marker (ALT, AST, ALP) levels, consistent with previous results of non-alcoholic fatty liver disease in db/db mice [67]. Surprisingly, CCL3 therapy improved hepatocyte steatosis and ballooning while lowering ALT levels. This shows that CCL3 may have hepatoprotective properties in addition to being non-toxic. These findings are consistent with reports that restoring prompt neutrophil responses can avoid further tissue harm by encouraging early clearance of necrotic material and minimizing persistent inflammation [68,69].

Histological evaluation is an essential element of toxicity testing because it provides a direct, observable, and detailed view of a substance’s effect on tissues and cells. Histopathological examination revealed that db/db mice had characteristic diabetes-associated lesions in multiple organs, including hepatic steatosis, hepatocyte ballooning, tubular necrosis in the kidneys, white pulp disruption in the spleen, pancreatic islet degeneration, and focal myofibrillar injury in cardiac tissue. These findings are consistent with previous reports describing multisystem injury in leptin receptor-deficient diabetic mice. Chronic hyperglycemia drives oxidative stress, inflammation, and structural organ damage [70,71]. Importantly, topical CCL3 did not exacerbate these pathological features; instead, organ scores in CCL3-treated groups were comparable to those in PBS-treated diabetic mice, confirming the absence of systemic organ toxicity. This distinguishes CCL3 from several proinflammatory chemokines, which, if dysregulated, can cause tissue injury [72].

CCL3 has also been shown to reduce infection and promote wound healing in nondiabetic mice [33], underscoring its potential as a novel therapeutic approach in wound care. Given the critical role of neutrophils in host defense, immunomodulatory strategies that enhance neutrophil mobilization and activation at sites of infection can be highly effective in controlling infection while simultaneously promoting tissue repair and healing [33,73,74,75,76,77,78].

Although repeated dosing is commonly used in clinical wound care, this study was specifically designed to evaluate the cytotoxicity associated with a single topical administration of CCL3. This approach was informed by our prior work demonstrating that transient restoration of early inflammatory signaling can be sufficient to reprogram the diabetic wound microenvironment and initiate a sustained healing response [4]. Nevertheless, differences between murine and human wound biology, as well as variability in clinical wound management, limit direct extrapolation of dosing frequency. Accordingly, the lack of repeated dosing studies represents a limitation of the present work, and future investigations will be required to determine whether a single-application strategy is adequate or whether repeated dosing confers additional efficacy or raises safety considerations in human diabetic wounds.

Collectively, these findings position CCL3 as a promising candidate biologic that addresses two critical aspects of diabetic wound care: compromised immune defense and safety. Unlike growth factors like recombinant PDGF-BB (Becaplermin), which showed limited efficacy and raised safety concerns, CCL3 appears to combine efficacy in infection control and wound repair with a good systemic tolerability profile. These findings meet a critical translational benchmark, as regulatory guidelines (FDA, ICH M3[R2], ICH S6[R1]) emphasize the importance of conducting preclinical safety studies in relevant disease models before moving biologics into clinical trials. Future research should build on these findings by examining chronic dosing regimens, assessing long-term immunogenicity, and testing in secondary infection models that mimic clinical wound contamination. Nonetheless, the current study provides a solid preclinical foundation, demonstrating efficacy and safety in the diabetic population.

## 5. Conclusions

This study shows that topical administration of CCL3 in diabetic mice is safe and well-tolerated, with no evidence of systemic organ toxicity. CCL3 reduced hepatocellular injury markers, preserved hematological and biochemical homeostasis, and did not cause abnormal immune responses. CCL3 accelerates wound closure in db/db mice, demonstrating a dual role in restoring impaired immune function and promoting tissue repair. These new safety studies highlight CCL3 as a promising biologic for diabetic wound therapy, combining immunomodulatory efficacy with a favorable systemic safety profile, and represent an important step toward clinical translation. In the future, we aim to further characterize the hepatoprotective activity and therapeutic relevance of CCL3.

## Figures and Tables

**Table 1:** Serum immunological parameters following 14-day topical CCL3 treatment in diabetic mice. Serum levels of IgG1, IgE, and C-reactive protein (CRP) were measured in nondiabetic control (db/+) mice treated with PBS and diabetic (db/db) mice treated with PBS, CCL3 (1 µg), or CCL3 (10 µg) on day 14. Data are expressed as mean ± SEM (*n* = 6 per group).

Parameters	Ctrl + PBS	db/db + PBS	db/db + CCL3 (1 µg)	db/db + CCL3 (10 µg)
**IgG1 (µg/mL)**	192.45 ± 13.51	285.75 ± 86.27	137.97 ± 21.18	198.86 ± 7.50
**IgE (µg/mL)**	94.29 ± 22.03	207.35 ± 57.64	150.41 ± 17.4	266.25 ± 51.61
**CRP (ng/mL)**	1.83 ± 0.33	8.14 ± 1.86	6.97 ± 1.66	8.29 ± 0.88

**Table 2:** Serum biochemical parameters following 14-day topical CCL3 treatment in diabetic mice. Serum biochemical indices were evaluated on day 14 in control (db/+) mice treated with PBS and diabetic (db/db) mice treated with PBS, CCL3 (1 µg), or CCL3 (10 µg). Parameters included liver function markers (ALT, AST, ALP), renal function markers (BUN, creatinine), electrolytes, glucose, and total protein. Data are expressed as mean ± SEM (*n* = 6 per group). Standard physiological ranges are shown for reference.

Parameters	Ctrl + PBS	db/db + PBS	db/db + CCL3 (1 µg)	db/db + CCL3 (10 µg)	Standard Range
**Alanine Transaminase U/L**	23.27 ± 1.33	275.65 ± 22.94	210.35 ± 11.43	211.18 ± 22.02	0–403
**Aspartate Transaminase U/L**	83.39 ± 6.18	247.58 ± 11.97	264.39 ± 14.76	209.20 ± 14.21	0–552
**Alkaline Phosphatase U/L**	97.93 ± 16.37	217.00 ± 6.32	191.98 ± 21.03	212.70 ± 27.02	49–172
**Blood Urea Nitrogen mg/dL**	26.97 ± 0.83	30.40 ± 1.10	31.05 ± 0.99	29.60 ± 1.39	15.2–34.7
**Calcium mg/dL**	10.17 ± 0.07	10.89 ± 0.18	10.52 ± 0.38	10.05 ± 0.40	9.6–11.5
**Chloride mmol/L**	114.92 ± 0.76	109.42 ± 0.61	109.13 ± 1.53	110.28 ± 2.03	105–118
**Creatinine mg/dL**	0.03 ± 0.00	0.06 ± 0.00	0.05 ± 0.01	0.05 ± 0.00	0.0–0.3
**Glucose mg/dL**	231.68 ± 24.41	402.48 ± 34.02	464.52 ± 27.46	477.40 ± 87.53	130–254
**Potassium mmol/L**	4.36 ± 0.10	5.06 ± 0.23	6.84 ± 1.05	6.11 ± 1.04	6.9–10.0
**Sodium mol/L**	155.33 ± 1.02	154.33 ± 0.92	155.50 ± 1.28	156.80 ± 3.32	150–160
**Phosphorus mg/dL**	9.98 ± 0.82	11.10 ± 0.62	14.03 ± 0.85	14.39 ± 1.35	7.5–10.7
**Total Bilirubin mg/dL**	0.05 ± 0.01	0.06 ± 0.01	0.06 ± 0.02	0.06 ± 0.01	0.0–0.2
**Total Protein g/dL**	4.71 ± 0.18	5.80 ± 0.11	5.01 ± 0.87	5.88 ± 0.31	4.7–6.1

**Table 3:** Hematological parameters following 14-day topical CCL3 treatment in diabetic mice. Hematological profiles, including white blood cell (WBC) count, red blood cell (RBC) indices, platelet count, and leukocyte differentials, were analyzed on day 14 in control (db/+) mice treated with PBS and diabetic (db/db) mice treated with PBS, CCL3 (1 µg), or CCL3 (10 µg). Data are expressed as mean ± SEM (*n* = 6 per group).

Parameters	Ctrl + PBS	db/db + PBS	db/db + CCL3 (1 µg)	db/db + CCL3 (10 µg)	Standard Range
**WBC (K/µl)**	9.89 ± 1.55	15.10 ± 0.79	9.46 ± 1.60	9.08 ± 1.28	5.1–14.7
**Hemoglobin (g/dL)**	10.77 ± 0.29	12.03 ± 0.54	12.33 ± 0.62	11.78 ± 0.37	11.7–16.2
**Neutrophil %**	17.48 ± 2.60	20.75 ± 1.34	16.79 ± 2.02	17.44 ± 0.91	12.5–31.2
**Lymphocyte %**	72.07 ± 3.84	68.00 ± 1.95	72.94 ± 2.81	73.16 ± 1.23	62.9–82.7
**Monocyte %**	6.32 ± 0.47	6.66 ± 0.27	5.55 ± 0.36	5.69 ± 0.37	2.5–7.5
**Eosinophil %**	3.33 ± 0.68	3.77 ± 0.46	3.56 ± 0.67	3.11 ± 0.13	1–3%
**Basophil %**	0.89 ± 0.16	0.89 ± 0.10	0.92 ± 0.11	0.58 ± 0.10	<1%
**Hemoglobin (g/dL)**	10.77 ± 0.29	12.03 ± 0.54	12.33 ± 0.62	11.78 ± 0.37	11.0–16.2
**MCV (fL)**	55.67 ± 2.90	58.33 ± 0.41	58.65 ± 1.06	58.78 ± 0.96	45.0–55.0
**Platelets (K/µL)**	757.67 ± 139.47	746.33 ± 67.57	813.67 ± 108.18	925.20 ± 95.57	574–1079
**MPV (fL)**	6.00 ± 0.15	6.10 ± 0.08	6.17 ± 0.12	6.00 ± 0.10	5.0–20.0

## Data Availability

Data supporting these findings can be found within the article.
